# Green Propolis Extract‐Mediated Synthesis of Biogenic Silver Nanoparticles: In Vitro Antileishmanial and Antibacterial Activities, Cytotoxicity and Ex Vivo Irritation Testing

**DOI:** 10.1002/cbdv.202402348

**Published:** 2025-05-20

**Authors:** Erica Tirzah S. Lima, Victoria L. S. Santos, Wanessa J. S. Mota, Frederico S. Martins, Ricardo L. C. de Albuquerque‐Junior, André L. S. Santos, Simone S. C. Oliveira, Jéssica A. de Lima, Adriana de Jesus Santos, Cochiran P. dos Santos, Sona Jain, Eliana B. Souto, Juliana C. Cardoso, Patrícia Severino

**Affiliations:** ^1^ Institute of Research and Technology, University Tiradentes Aracaju Brazil; ^2^ School of Pharmaceutical Sciences of Ribeirão Preto, University of São Paulo São Paulo Brazil; ^3^ Department of Pathology Federal University of Santa Catarina Florianópolis Brazil; ^4^ Department of General Microbiology Paulo de Góes Institute of Microbiology Federal University of Rio de Janeiro Rio de Janeiro Brazil; ^5^ Laboratory for the Advanced Study of Emerging and Resistant Microorganisms Federal University of Rio de Janeiro Rio de Janeiro Brazil; ^6^ Apis Flora, Rua Triunfo Ribeirão Preto Brazil; ^7^ Department of Physics and Materials Science and Engineering Federal University of Sergipe São Cristóvão Brazil; ^8^ Department of Morphology Federal University of Sergipe São Cristóvão Brazil; ^9^ UCD School of Chemical and Bioprocess Engineering University College Dublin Belfield Ireland

**Keywords:** antileishmanial, antimicrobial, green propolis, Hen's egg chorioallantoic membrane, silver nanoparticles

## Abstract

This study describes the green synthesis, characterization, and biological evaluation of silver nanoparticles (AgNPs) obtained from green propolis (AgNPs‐PRO). Before nanoparticles synthesis, a hydroethanolic green propolis extract (GPE) was obtained through ultrasound‐assisted extraction and characterized by high‐performance liquid chromatography, revealing the artepilin C as the most abundant phenolic compound in its composition, followed by 4,5‐dicaffeoylquinic acid and drupanin. The analysis of synthesized AgNPs by UV–Vis spectroscopy showed a characteristic absorption band at 430 nm. Dynamic light scattering analysis revealed mean hydrodynamic particle sizes ranging from 88 to 115 nm, with a polydispersity index between 0.229 ± 0.006 and 0.365 ± 0.054. Fourier‐transform infrared spectroscopy confirmed that functional groups present in GPE contribute to the reduction and stabilization of AgNPs. Differential scanning calorimetry and transmission electron microscopy confirmed that AgNPs were obtained. GPE showed leishmanicidal activity against promastigote forms of *Leishmania amazonensis*, with a half‐maximal inhibitory concentration (IC_50_) of 11.87 µg/mL and a selectivity index (SI) of 12.52. Antibacterial activity of the AgNPs, assessed via the disk diffusion method, revealed inhibition zones against *Escherichia coli* (Gram‐negative), *Staphylococcus aureus* (Gram‐positive), and *Candida albicans* strains. The HET‐CAM test indicated no signs of irritation, suggesting the biocompatibility of the developed AgNPs.

## Graphical abstract

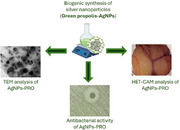

## 1 Introduction

The biological synthesis of silver nanoparticles (AgNPs) is characterized by its simplicity, rapid production, low toxicity, and environmental friendliness. The obtained AgNPs show important properties, such as high yield and stability, and have a wide range of potential applications as catalysts in biomedical imaging and drug delivery [1, 2, 3, 4]. Biological materials used in the synthesis of AgNPs include natural extracts and compounds derived from fungi and bacteria [5, 6, 7].

Natural extracts obtained from green propolis are resinous substances from plant buds and exudates collected by bees. They are distinguished from other plant extracts by their unique composition and potent biological activities, making them an ideal candidate for biomedical and pharmaceutical applications requiring natural antioxidants and antimicrobial agents. Unlike many other plant extracts, green propolis contains a high concentration of artepillin C, a phenolic compound known for its potent anti‐inflammatory, antimicrobial, and antitumor properties. Studies have shown that the antioxidant capacity of green propolis exceeds that of other commonly used plant extracts rich in phenolic compounds and flavonoids, such as green tea and grape seed [8]. Green propolis also has a well‐documented history of use in traditional medicine, particularly in regions like Brazil, where its healing properties have been exploited for centuries. These factors, combined with bioavailability and stability, make green propolis a superior choice for natural product development for various therapeutic uses [9]. Green propolis contains potent reducing agents that facilitate the reduction of silver ions (Ag^+^) to AgNPs. The mechanism involves the donation of electrons from these bioactive compounds, which reduces Ag^+^ ions to Ag^0^, thereby forming AgNPs. This bioreduction process is central to this study, as it highlights the natural and efficient pathway through which AgNPs are synthesized [10].

The antimicrobial activity of AgNPs is mainly attributed to their physicochemical properties, such as average size, size distribution and shape, in addition to concentration. The mechanism of action has been linked to several factors, including damage to the cell membrane of bacteria or the plasma membrane of fungi. This mechanism leads to the loss of cellular components, disruption of the respiratory chain, and synthesis of adenosine triphosphate (ATP), which affects the cellular energy source, causing death of the microorganism, damage to deoxyribonucleic acid (DNA) and disruption of cell replication [11, 12]. The use of AgNPs in consumer products, such as in cosmetics and medical applications, has gained considerable attraction due to their well‐documented efficacy against bacteria, fungi [13, 14], and even protozoa, such as *Toxoplasma gondii* [15] *Schistosoma mansoni* [15], and *Leishmania amazonensis* [16].

To ensure the safety of biosynthesized AgNPs as antimicrobial and antifungal agents, it is necessary to assess their cytotoxicity in, for example, macrophages, and also their potential risk of irritation. As key players in the immune system, macrophages have a crucial role in determining the safety of AgNPs for therapeutic applications for leishmaniasis treatment [17]. These evaluations are critical to validate the biocompatibility of nanoparticles and to mitigate potential adverse effects, thereby ensuring their safety and therapeutic efficacy in clinically sensitive contexts such as wound healing and the treatment of cutaneous infections.

## 2 Materials and Methods

### 2.1 Materials

Silver nitrate (AgNO_3_, 99.9%) was purchased from Química Contemporânea (Indaiatuba, São Paulo, Brazil), and all the other reagents were bought from Sigma‐Aldrich (St. Louis, MO, USA). Crude green propolis was kindly donated by the Federal University of Santa Catarina (Florianopolis, Santa Catarina, Brazil) and the filtered double distilled water was home supplied (Milipore, Millipore GmbH, Burlington, MA, USA).

### 2.2 Preparation and Characterization of Green Propolis Extract

The raw samples of green propolis were ground using a mortar and pestle, stored in tightly sealed containers, and kept in a freezer until ultrasound‐assisted extraction (UAE). This technique employs ultrasound to enhance extraction efficiency by disrupting cell walls to improve solvent penetration, thus maximizing the extraction yield of bioactive compounds from the propolis. The green propolis extract (GPE) was prepared according to the protocol described by Loureiro et al. [18]. Briefly, 5 g of green propolis were dissolved in 25 mL of 70% ethanol and sonicated for 1 h at 25°C (Ultra Cleaner 1400A, Unique Brazil, Fortaleza, Brazil). Subsequently, the extract was subjected to centrifugation at 1800 × *g* for 15 min using a Hettich benchtop centrifuge Rotina 380 (Berlin, Germany). The obtained samples were held at room temperature in a fume hood for 24 h under air exposure for subsequent evaporation of the supernatant.

The yield of extraction, indicating the solvent's efficacy in isolating certain components, was determined by the mass of extract obtained (*M*_f_) from the original mass (*M*_i_) of the raw material, represented as a percentage, using the following equation:

$$\mathrm{Yield}\;\mathrm{of}\;\mathrm{extraction}\;\left(\%\right)=\frac{M_{f}}{M_{i}}\times 100$$

The high‐performance liquid chromatography (HPLC) analysis was performed in a Shimadzu (Kyoto, Japan), with a CBM‐20A controller, LC‐20AT quaternary pump, SPD‐M 20A diode array detector, and recording the data in a Shimadzu LC software version 1.21 SP1. Chromatographic separation was achieved using a Shimadzu Shim‐Pack CLC‐ODS column (4.6 mm × 250 mm, particle size 5 µm, pore size 100 Å). The mobile phase comprised methanol (B) and acidified water containing formic acid (0.1% v/v) (A). The technique employed a linear gradient from 20% to 95% B for a duration of 77 min at a flow rate of 0.8 mL/min. The injection volume was 10 µL, and the column oven temperature was maintained at 40°C. Detection was carried out at 275 nm.

### 2.3 Biogenic Synthesis of AgNPs

To determine the optimal conditions for the biogenic synthesis of AgNPs with green propolis (AgNP‐PRO) based on particle size, the concentration of AgNO_3_ varied at three levels (1, 3, and 5 mM). Based on the determined parameters, the propolis extracts were resuspended at a concentration of 0.5 mg/mL and added to water under stirring at 650 rpm on a K40‐182OH (Kasvi, Pinhais, Paraná, Brazil) in a magnetic stirrer plate for 30 min. AgNO_3_ was then added and, after 30 min, the resulting colloidal suspension was stored away from light. The experiments were conducted at 25 ± 2°C.

### 2.4 Particle Size Analysis and Kinetics of Nanoparticle Formation

Dynamic light scattering (DLS) was used to determine the mean particle size in a Zetasizer Nano ZS90 (Malvern, England), employing a refractive index of 1.390 and an absorption coefficient of 0.002 across 20 runs. DLS provides insights into the hydrodynamic diameter and polydispersity index (PDI), ensuring accurate measurement of nanoparticle size distribution and shelf stability. To monitor the kinetics of nanoparticle formation under the selected conditions, nanoparticle absorption was measured in a UV–Vis spectrophotometry using a plate reader (Flextation 3, Sunnyvale, CA, USA) in the wavelength range of 200–800 nm. UV–Vis spectrophotometry tracks the surface plasmon resonance (SPR), providing real‐time data on nanoparticle formation and growth.

### 2.5 Fourier‐Transform Infrared Spectroscopy

Fourier‐transform infrared (FTIR) analysis was conducted to identify the functional groups present in the material, covering a range of 400–4000 cm^−1^, using an Agilent Technologies Cary 630 FTIR spectrometer (Santa Clara, CA, USA). This technique allows for the detection of characteristic molecular vibrations, providing key information about the chemical bonds and functional groups on the nanoparticles' surface, which are crucial for understanding nanoparticles' interactions and stability.

### 2.6 Differential Scanning Calorimetry

The calorimetric analysis of the AgNPs (AgNPs‐PRO) synthesized with GPE was performed in a DSC‐60 (Shimadzu, Kyoto, Japan). The analysis was conducted over a temperature range from 25°C to 600°C, with a heating rate of 5°C/min, under a nitrogen atmosphere with a flow rate of 45 mL/min. Differential scanning calorimetry (DSC) provides thermal data on the nanoparticles, such as phase transitions, stability, and decomposition, which are essential for evaluating the thermal behavior and crystallinity of the AgNPs.

### 2.7 Transmission Electron Microscopy

A JEOL transmission electron microscope (TEM‐MSC JEOL 210, Tokyo, Japan) was used to confirm the synthesis of AgNPs. A volume of 20 µL of nanoparticles was deposited onto a carbon film‐coated cooper grid for 2 min, followed by negatively staining with uranyl acetate (2% m/v) for another 2 min and then left to dry under air exposure. Electron diffraction analysis of the selected area (SAED) of the particles was recorded using the microscope software.

### 2.8 Parasites and Cultivation

*L. amazonensis* strain (MHOM/BR/PH8) was obtained from the Coleção de Leishmania of the Fundação Oswaldo Cruz (FIOCRUZ; Leishmania Type Culture Collection‐LTTC‐WDCM 731, Rio de Janeiro, Brazil). Promastigotes were cultivated in Schneider's insect medium (Sigma‐Aldrich, St Louis, MO, USA), pH 7.2, supplemented with 10% heat‐inactivated fetal bovine serum (FBS) (Cultilab, São Paulo, SP, Brazil) at 28°C.

### 2.9 Macrophages Cultivation

The human leukemia monocytic cell line (THP‐1), obtained from American Type Culture Collection (ATCC, Manassas, VA, USA), was grown in 25 cm^2^ tissue culture flasks using RPMI 1640 media (Sigma‐Aldrich, St Louis, MO, USA) supplemented with 10% FBS at 37°C in a 5% CO_2_ atmosphere. The culture medium was replaced every three days. In interaction experiments, THP‐1 cells (2 × 10^5^ cells/well) were cultured in 96‐well plates and differentiated into macrophages through treatment with phorbol‐12‐myristate‐13‐acetate (PMA; 40 ng/mL) (Sigma‐Aldrich, St Louis, MO, USA) for a duration of 48 h. The plates were subsequently rinsed twice with sterile phosphate‐buffered saline (PBS; pH 7.2) to eliminate PMA, after which a fresh RPMI 1640 medium was introduced.

### 2.10 Effects of GPE on Promastigotes' Growth Rate

Promastigotes were quantified using a Neubauer chamber and resuspended in fresh Schneider's insect medium to achieve a final concentration of 5 × 10^5^ viable promastigotes per milliliter. Viability was determined based on motility and the absence of staining following exposure to Trypan blue (Sigma‐Aldrich, St. Louis, MO, USA). The GPE was added to the cultures. These were prepared from a stock solution in dimethyl sulfoxide (DMSO; Sigma‐Aldrich, St. Louis, MO, USA). After 72 h of incubation at 28°C, the number of viable parasites was assessed. The 50% inhibitory concentration (IC_50_), defined as the concentration of the compound that resulted in a 50% reduction in parasite viability, was calculated through linear regression analysis. This analysis involved plotting the logarithm of the number of promastigotes against the drug concentration using GraphPad Prism 5 software [19].

### 2.11 Macrophage Toxicity

The effects of GPE on the viability of THP‐1 cells were evaluated using the (3‐(4,5‐dimethylthiazolyl‐2)‐2,5‐diphenyltetrazolium bromide) (MTT) assay. THP‐1 cells (2 × 10^5^ cells/mL) were differentiated, as described in Section 2.9, in 96‐well culture plates in RPMI 1640 medium supplemented with 10% FBS. Then, the GPE was added to the cultures at specific final concentrations of 5, 10, 15, 20, and 25 µg/mL, and the macrophage cells were incubated in a biochemical oxygen demand (BOD) incubator (MRC Global, Houston, TX, USA) for 24 h at 37°C in a 5% CO_2_ atmosphere. The 50% cytotoxic concentration (CC_50_) was determined by a linear regression analysis after 24 h of treatment with GPE.

### 2.12 Disc Diffusion Test

The Kirby‐Bauer disc diffusion method was utilized to assess the antimicrobial activity of AgNP‐PRO. Mueller–Hinton agar plates were employed along with Whatman No. 1 filter paper discs (Sigma‐Aldrich, St. Louis, MO, USA). To determine effective concentration, 40 µL of sodium borohydride (NaBH_4_), AgNP‐PRO at concentrations of 1, 3, and 5 mM, and GPE were tested using the disc diffusion method. Representative microorganisms tested were obtained from ATCC (Manassas, VA, USA) and included *Staphylococcus aureus* (ATCC 29213), *Escherichia coli* (ATCC 25922), and *Candida albicans* (ATCC 90028). From an overnight microbial culture, four to five colonies were picked up using a sterilized inoculating loop and emulsified in 5 mL of sterile normal saline to achieve turbidity equivalent to McFarland No. 0.5. A sterile swab was immersed in the microbial suspension, and any surplus fluid was removed by pressing it against the tube's wall. The surface of a Mueller–Hinton agar plate was inoculated with the microbial isolate, with the plate being rotated 90° and streaked for even distribution. Discs impregnated with 40 µL of NaBH_4_, AgNP1‐PRO, AgNP3‐PRO, AgNP5‐PRO, and GPE were placed on the agar plates. The plates were incubated at 37°C for 16 h. Following incubation, the plates were examined to determine the size of the inhibition zones. The antimicrobial activity was evaluated by measuring the zones of inhibition using ImageJ software [19].

### 2.13 Hen's Egg Chorioallantoic Membrane Test

Fertilized chicken eggs were obtained from a local producer (Fazenda Asa Branca, São Cristóvão, Brazil) immediately post‐laying and incubated in an automatic rotating incubator for 10 days under regulated conditions of temperature (37.8 ± 1.0°C) and relative humidity (45%–65%). On Day 10, the eggs were candled to assess embryo viability, and any abnormal eggs were eliminated. The shell was subsequently excised with forceps at the air cell, and the inner membrane was carefully detached to expose the highly vascularized chorioallantoic membrane (CAM). The irritation potentials of AgNP‐PRO, prepared with varying concentrations of AgNO_3_, NaBH_4_, and GPE, were assessed using the Hen's egg test on the chorioallantoic membrane (HET‐CAM). Following exposure to 0.2 mL of each test sample, 0.9% saline (NaCl) solution (negative control), or a 0.1 mol/L sodium hydroxide (NaOH) solution (positive control), the CAMs were carefully washed with 0.9% saline solution [20]. Vascular responses, including hemorrhage (*H*), coagulation (*C*), and vessel lysis (*L*), were checked and documented using a camera. The irritation potentials were evaluated based on the severity of irritation scores (IS scores), categorized as nonirritant (IS < 6), mildly irritant (6 ≤ IS < 12), moderately irritant (12 ≤ IS < 15), and severely irritant (IS ≥ 15), and determined using the following equation [21]:

$$300\left[\mathrm{IS}\right]=5\left[301-H\right]+7\left[301-L\right]+9\left[301-C\right]$$

where *H* is the start time in seconds for the onset of bleeding, *L* is the start time in seconds for lysis, and *C* is the start time in seconds for coagulation. The IS scores reflect the highest total score recorded for any of the three endpoints (hemorrhage, coagulation, and vessel lysis) monitored for 300 s, as described by Barbosa et al. [20].

### 2.14 Statistical Analysis

All experiments were performed in triplicate, and the data were expressed as mean ± standard deviation, with one‐way analysis of variance (ANOVA) followed by Tukey's test (*p* ≤ 0.05).

## 3 Results and Discussion

### 3.1 Extract Yield Percentage and TPC Analysis

UAE was employed to obtain the bioactive compounds from green propolis, resulting in a yield of 37 ± 0.46%. This value aligns with the results reported by Oroian et al. [19] for red propolis, demonstrating the efficiency of this technique. Notably, the yield obtained exceeded the minimum dry extract content of 11% recommended by the Brazilian Ministry of Agriculture [22], confirming the method's suitability for extracting compounds from green propolis. The total phenolic content (TPC) of the green propolis hydroethanolic extract was 150.93 mg GAE/g, determined using a gallic acid calibration curve (*R* ^2^ = 0.9996). This value indicates a high level of bioactive compounds and aligns with Frozza et al. [23], who reported 152 mg GA/g for red propolis. Moncayo Luján et al. [24] found 21.22 mg GA/g in Mexican propolis extracted with 70% ethanol via ultrasound. In China, ultrasound‐extracted propolis showed TPCs ranging from 72 to 201 mg GA/g [25], reflecting regional and methodological variations.

The enhanced performance of GPE is linked to its high content of flavonoids, phenolics, and caffeic acid derivatives, which act as potent antioxidants. These compounds reduce Ag^+^ to Ag^0^ via electron donation, promoting efficient AgNP synthesis. In addition, they stabilize the nanoparticles by forming a capping layer, minimizing aggregation and improving shelf‐life stability crucial for applications in medicine and environmental science [26].

### 3.2 Chromatographic Analysis

**FIGURE 1:**
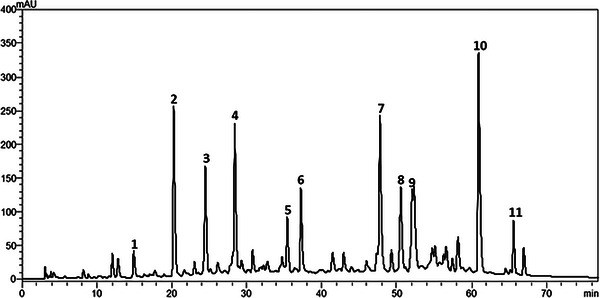
High‐performance liquid chromatography (HPLC) of the hydroethanolic extract of green propolis. Milliunits of absorbance (mAU) as a function of retention time (min) of phenolic compounds.

Figure 1 shows peaks with retention times similar to the standards, between 10 and 70 min, indicating more polar constituents. This region reveals four distinct peaks identified as caffeic acid (1), *p*‐coumaric acid (2), 3,5‐dicaffeoylquinic acid (3), 4,5‐dicaffeoylquinic acid (4), cinnamic acid (5), aromadendrin (6), drupanin (7), chrysin (8), galangin (9), artepillin C (10), and baccarin (11). The peaks were assigned on the basis of the retention times. All 11 analyzed compounds were identified and quantified in the extract (Table 1). Identifying and quantifying bioactive compounds in propolis is necessary since each type of propolis has unique characteristics that can be exploited for specific therapeutic applications [16, 17].

**TABLE 1:** Concentrations of phenolic compounds found in the hydroethanolic extract of green propolis through high‐performance liquid chromatography (HPLC).

Number	Chemical compound	Concentration (mg/g)
1	Caffeic acid	2.325 ± 0.023
2	*p*‐Coumaric acid	9.1 ± 0.06
3	3,5‐Dicaffeoylquinic acid	16.357 ± 0.16
4	4,5‐Dicaffeoylquinic acid	30.187 ± 1.08
5	Cinnamic acid	1.408 ± 0.01
6	Aromadendrin	10.977 ± 0.120
7	Drupanin	20.441 ± 0.41
8	Chrysin	4.638 ± 0.0474
9	Galangin	6.742 ± 0.271
10	Artepillin C	45.717 ± 3.48
11	Bacharidin	3.039 ± 0.15

In our study, the three most abundant phenolic compounds identified in the hydroethanolic extract of green propolis were artepilin C (45.71 mg/g), 4,5‐dicaffeoylquinic acid (30.18 mg/g), and drupanin (20.44 mg/g). These high concentrations indicate the effectiveness of the ultrasound extraction method in preserving and concentrating bioactive compounds.

Cinnamic compounds identified in the extract may contribute to its anti‐inflammatory, antidiabetic, anticancer, and antioxidant activities [27, 28, 29, 30]. Notably, these compounds may play a crucial role in macrophage activation, suggesting potential anti‐leishmania and antimicrobial effects. Aromadendrin, a flavonoid with reported anticardiac hypertrophy, antioxidant, anti‐inflammatory, and antidiabetic properties [31], was present at approximately 11 mg/mL.

### 3.3 UV–Vis Analysis

**FIGURE 2:**
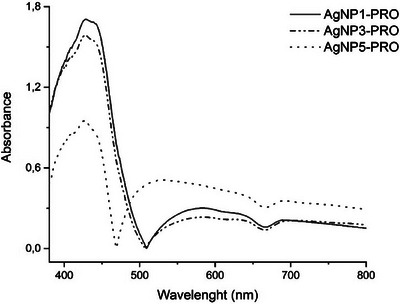
Absorption spectrum of the solution of silver nanoparticles biosynthesized with green propolis extract AgNP1‐PRO, AgNP3‐PRO, AgNP5‐PRO.

AgNPs exhibit absorption characteristics in the visible region of the spectrum, referred to as SPR. This spectrum provides insights into the size and shape of the particles obtained by exciting electromagnetic waves (plasmons) on the surface, suggesting a spherical form for these AgNP‐PRO in the wavelength range of 400–470 nm [32]. For the UV–Vis spectrophotometry analysis, three solutions of AgNO_3_ were prepared at concentrations of 1, 3, and 5 mM (Figure 2). In this study, all samples exhibited distinct SPR bands around 430 nm. The average nanoparticle sizes were determined as follows: 115.3 ± 0.71 nm for 1 mM, 109.4 ± 1.12 nm for 3 mM, and 88.48 ± 3.82 nm for 5 mM. These size variations align with the observed SPR bands, facilitating a comparison with existing literature on the relationship between UV peaks and nanoparticle size. Lower concentrations of AgNO_3_ resulted in the formation of more nanoparticles, leading to increased absorption intensity. Conversely, higher concentrations of AgNO_3_ led to fewer nanoparticles. Thus, AgNO_3_ concentration affects both the quantity and size of the AgNPs formed [33].

The SPR band also plays a critical role in assessing the stability of the nanoparticles. Shifts in the SPR peak or the appearance of new peaks at longer wavelengths can indicate nanoparticle aggregation. In examining various types of propolis, AgNPs displayed different average sizes, that is, 109 nm with a maximum intensity at 412 nm for red propolis [10], 13.09 nm with a peak at 428 nm for Indian propolis [32], 108 nm with a peak at 420 nm [34], and 40 nm with a peak at 424 nm for green propolis [26].

### 3.4 DLS Analysis

The DLS results of this study showed particle diameters ranging from 88 to 115 nm (Table 2). However, for the 5 mM concentration, a larger standard deviation of ±3.82 was obtained, indicating greater variability in size distribution. This suggests that higher AgNO_3_ concentrations result in less uniform nanoparticles, potentially due to increased instability during the synthesis process.

**TABLE 2:** Results of the DLS analysis of AgNPs with different AgNO_3_ concentrations.

Samples	Z‐average (nm)	PDI
AgNP1‐PRO	115.3 ± 0.71	0.339 ± 0.004
AgNP3‐PRO	109.4 ± 1.12	0.229 ± 0.006
AgNP5‐PRO	88.48 ± 3.82	0.365 ± 0.054

*Note*: Data are presented as the mean ± standard deviation.

Abbreviations: PDI, polydispersity index; Z‐average, mean particle size.

The PDI ranges from 0 to 1 and indicates the degree of nanoparticle distribution in a dispersion. Values equal to or below 0.3 indicate monodispersed particles without aggregation, while values above 0.3 indicate polydispersity [35]. In drug delivery studies, PDI values between 0.1 and 0.4 are generally acceptable, indicating a uniform nanoparticle size distribution. This homogeneity is crucial for ensuring formulation stability, reproducible drug release, and enhanced therapeutic efficacy [36].

As the concentration of AgNO_3_ increased, the particle sizes decreased. UV–Vis spectroscopy showed that larger particles (115 nm) in AgNP1‐PRO and (109 nm) in AgNP3‐PRO had higher intensity peaks, while smaller particles (88 nm) in AgNP5‐PRO exhibited lower absorbance intensity. Higher concentrations of AgNO_3_ resulted in the formation of two distinct peaks, likely due to increased agglomeration, as highlighted by a higher PDI value of 0.365.

Nanoparticles synthesized with Egyptian propolis showed hydrodynamic sizes of about 100 nm [32]. Similar results were observed in studies using AgNPs reduced and stabilized with different types of propolis, including white and red propolis, where the average size and PDI values were 108 and 109 nm, and 0.224 and 0.207 [34], yet not statistically significant (*p* > 0.05). Nevertheless, based on the results, a 3 mM concentration of AgNO_3_ produced smaller average particle sizes compared to the 1 mM concentration, while also achieving a lower PDI. This property may be desirable to ensure longer‐term stability on the shelf and uniform effects.

Studies highlight that the size and stability of AgNPs are influenced by the concentration and type of biogenic agents used. Akpobolokemi et al. [36] observed that lower concentrations of *Spinacia oleracea* leaf extract (2% and 3%) resulted in larger AgNPs (173 and 311 nm), while higher concentrations (4%, 7%, and 10%) reduced particle sizes to 148, 120, and 109 nm, respectively. Similarly, Jayachandran et al. [37] synthesized AgNPs with *Tabebuia pallida* leaf extract, obtaining a mean size of 172.1 nm and a PDI of 0.381.

### 3.5 FTIR Analysis

**FIGURE 3:**
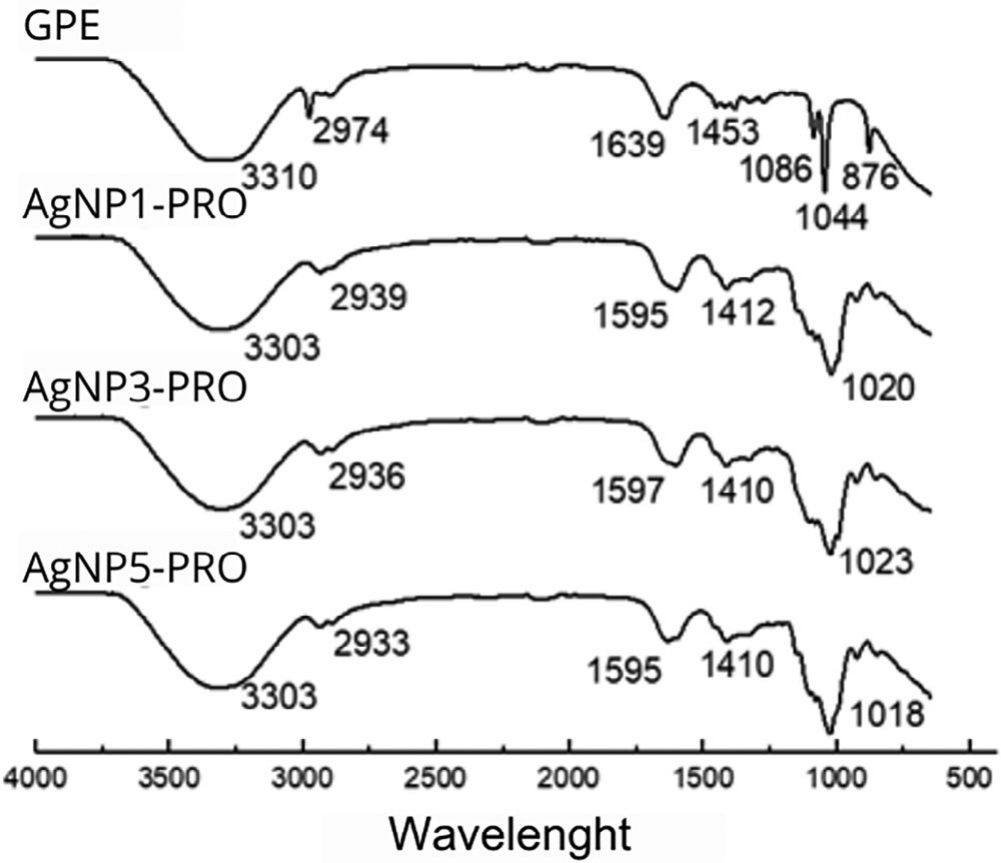
FTIR spectra of green propolis extract and AgNPs‐PRO.

FTIR spectra of GPE and PRO‐AgNPs can be visualized in Figure 3, respectively. In the propolis extract, the observed central bands at 3419 cm^−1^ were assigned to hydroxyls, at 1637 cm^−1^ representing the carbonyls and carboxyl, at 1380 and 1100 cm^−1^ are related to heterocyclic compounds (C─O─C) found in alkaloids and flavones, and at 831 cm^−1^ are related to the presence of glycosides [6].

Functional groups, such as carboxylates, hydroxyls, and amines, played an important role in the reduction and stabilization of AgNPs and were recorded, respectively, at 2941, 1602, and 1018 cm^−1^. A band displacement was observed at 3310 cm^−1^, corresponding to the hydroxyl region. The carbonyl and carboxyl groups exhibited a little decrease in intensity, indicating their involvement in bond formation with the peptide bands, as anticipated, since these groups are constituents of phenolic compounds and are integral parts of propolis. Finally, there is also the displacement of the region of heterocyclic compounds to 1018 cm^−1^, as well as the disappearance of the band referring to the glycosides, even suggesting the participation of these compounds in the reduction of silver for AgNP‐PRO formation [6].

### 3.6 DSC

**FIGURE 4:**
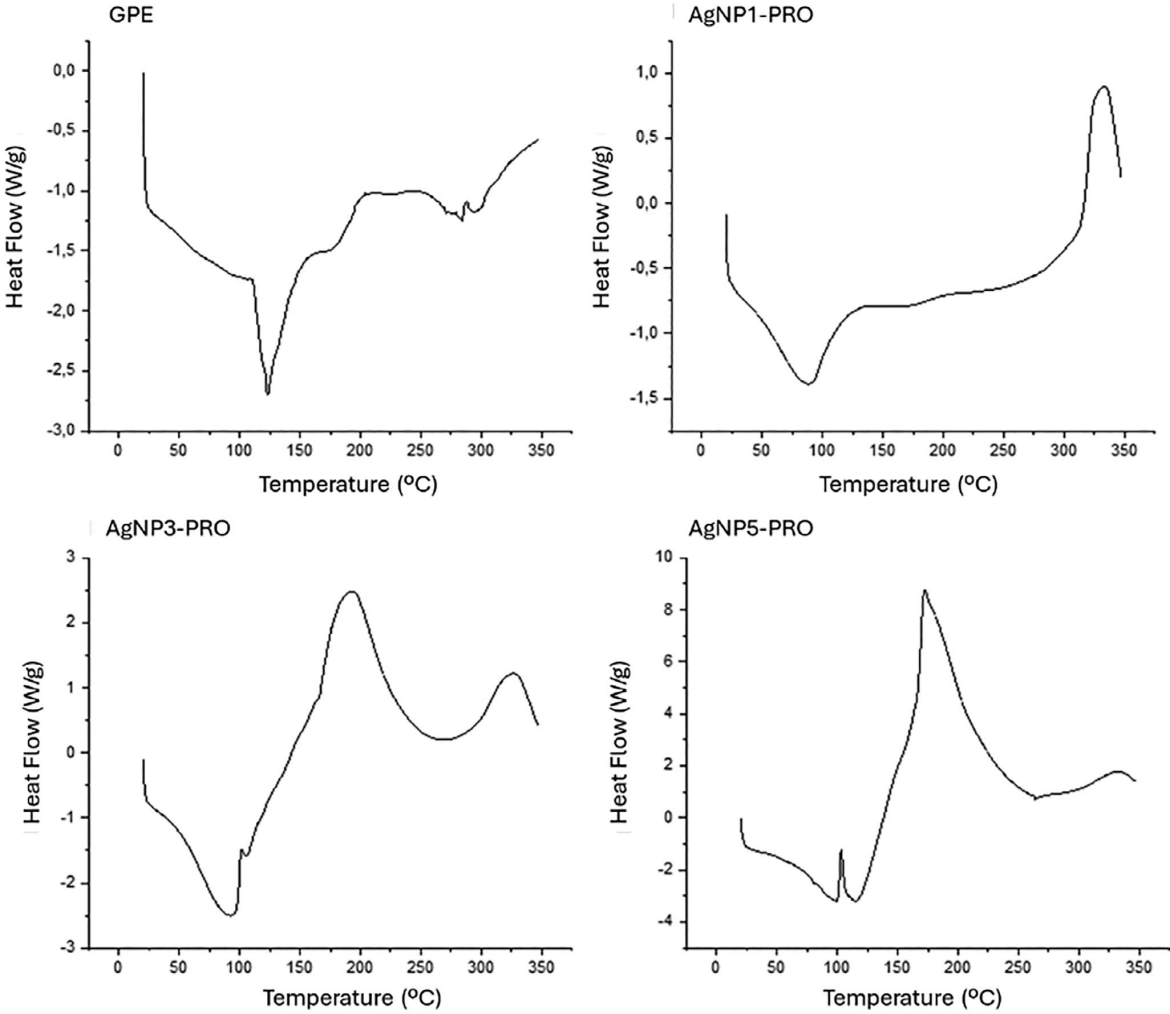
Thermoanalytical curves from the DSC analysis of the green propolis extract (GPE) and AgNPs coated with GPE using different AgNO_3_ concentrations: 1, 3, and 5 mM (AgNP1‐PRO, AgNP3‐PRO, and AgNP5‐PRO, respectively).

The DSC curves observed in GPE and AgNP‐PRO are shown in Figure 4, highlighting both endothermic and exothermic peaks associated with the physical and chemical changes in the samples. The thermoanalytical curve of GPE showed a significant endothermic peak at 123.32°C, indicating the melting of low molecular weight compounds, such as flavonoids and other phenolic compounds present in the extract. A smaller exothermic peak was observed around 300°C, associated with the decomposition of the extract.

Similar findings, which corroborate our results, were reported by Roy et al. [6] in their study with red propolis dry extract, where a peak around 145°C was assigned to the melting of compounds in the propolis extract, with decomposition occurring around 300°C.

The thermoanalytical curves of AgNP1‐PRO, AgNP3‐PRO, and AgNP5‐PRO displayed endothermic peaks at 88°C, 92°C, and 99°C, respectively. Exothermic events were observed at 193.11°C for AgNP3‐PRO and 172.66°C for AgNP5‐PRO, while no such event was detected in this range for AgNP1‐PRO. The exothermic peaks were identified between 300°C and 350°C at 327.88°C, 326.73°C, and 336.13°C for AgNP1‐PRO, AgNP3‐PRO, and AgNP5‐PRO, respectively. These findings are consistent with the study by Roy et al. [6], who investigated red propolis as a coating agent for nanoparticles and identified an endothermic event at 135°C, possibly associated with complex events, such as solvent volatilization or the melting of fats, waxes, and phenolic compounds. The exothermic events around 300°C are likely linked to the decomposition of silver oxide in the AgNPs. This process occurs as the sintering gradually causes the silver oxide to decompose, leading to the partial fusion of the particles.

### 3.7 Transmission Electron Microscopy

**FIGURE 5:**
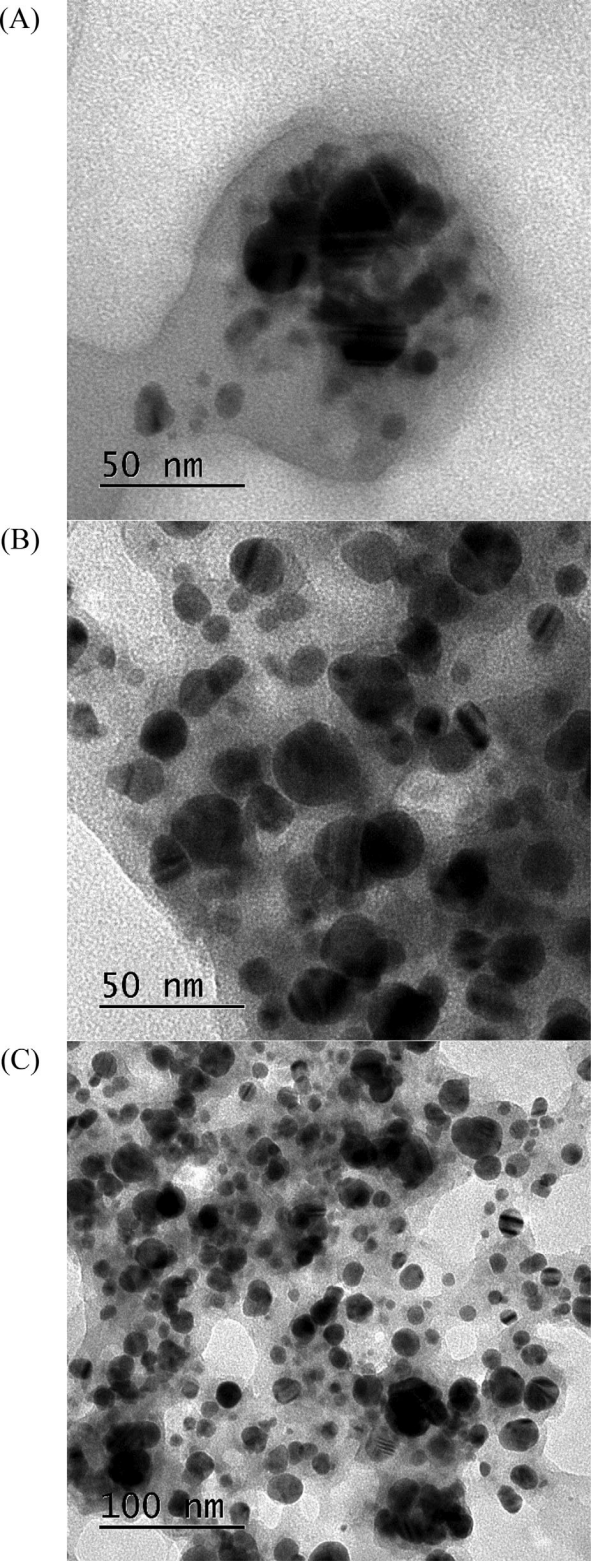
Transmission electron microscopy images of AgNPs synthesized with green propolis extract with (A) 1 mM of AgNO_3_ AgNP1‐PRO, (B) 3 mM of AgNO_3_ AgNP3‐PRO, and (C) 5 mM of AgNO_3_ AgNP5‐PRO) (265 000×).

The morphology of synthesized AgNPs is shown in Figure 5, confirming that individualized particles were obtained with GPE. The particle size recorded by transmission electron microscopy (TEM) was lower than DLS, since this latter gives the mean hydrodynamic diameter, that is, the diffusion layer surrounding the particle core, whereas TEM provides the core diameter of the particles under the field microscope. Figure 5 confirms that the highest AgNO_3_ concentration (5 mM) led to a lower size of particles (AgNP5‐PRO).

### 3.8 Effect of GPE on Parasite Proliferation

**FIGURE 6:**
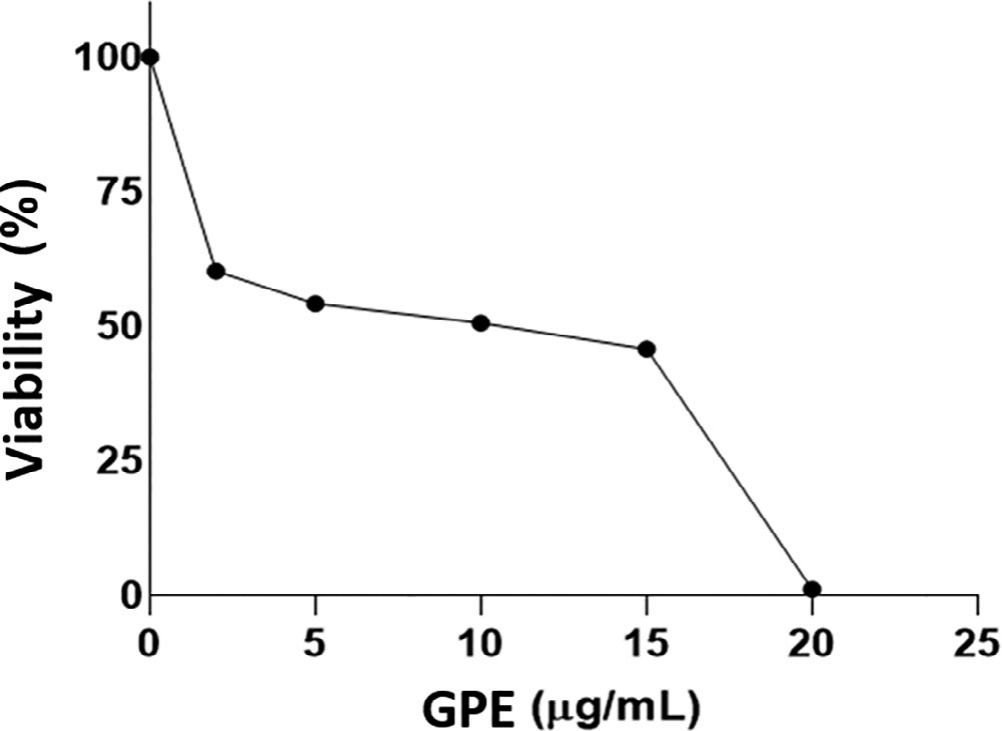
Leishmanicidal activity of green propolis extract (GPE) on *Leishmania amazonensis* promastigotes.

The leishmanicidal activity was assessed by determining the minimum amount of substance required to inhibit the growth of the microorganism, as measured by the IC_50_. Figure 6 illustrates the evaluation of the leishmanicidal activity of GPE against *L. amazonensis* promastigotes. This assessment was conducted to evaluate the extract used in biosynthesis and the subsequent analysis of the nanoparticles. The results demonstrated the efficacy of the GPE against *L. amazonensis* promastigotes, with an IC_50_ value of 11.87 µg/mL.

Given the central role of macrophages in infection, the THP‐1 cell line was used to assess the cytotoxicity of GPE, which demonstrated a favorable safety profile with a CC_50_ of 148.66 µg/mL. Compared to conventional drugs like amphotericin B and meglumine antimoniate, GPE exhibited greater potency at lower concentrations, as indicated by its lower IC_50_. In addition to directly inducing oxidative stress in parasites, GPE also modulates host immune responses, offering a dual mechanism that may enhance therapeutic efficacy and reduce the likelihood of resistance development.

Recent studies have demonstrated the selective cytotoxicity of various nanomaterials towards cancer cells, while maintaining relative safety for normal cells. Khalil et al. [38] reported that ZnO‐NPs exhibited higher cytotoxicity against CAL27 cancer cells (IC_50_ = 52.15 µg/mL) compared to normal HFB4 fibroblasts (IC_50_ = 36.3 µg/mL), indicating preferential toxicity of ZnO‐NPs toward malignant cells. Tanka et al. [39] showed that AgNPs at 20 mM exhibited minimal cytotoxicity toward Vero cells, with CC_50_ = 60.52 µg/mL. These findings highlight the importance of concentration, formulation, and cellular context when evaluating nanomaterial biocompatibility.

The high selectivity index (SI) of GPE highlights its favorable safety profile, with lower host cell cytotoxicity compared to the nephrotoxic effects of amphotericin B and other antimonials. In addition, the nanoparticle size allows for targeted delivery and sustained release, enhancing therapeutic efficacy. These properties reinforce the potential of GPE as a promising candidate for combination therapies, in particular, to overcome antimicrobial resistance. Overall, GPE offers a safer, more effective, and sustainable alternative for the treatment of leishmaniasis with remarkable clinical relevance (Table 3).

**TABLE 3:** Anti‐leishmanial activity against *Leishmania* promastigotes and selectivity index (SI) of GPE and reference leishmanicidal drugs.

Compounds	IC_50_ promastigotes (µg/mL)	SI	Reference
GPE	11.87	12.52	Present work
Amphotericin B	33.30	2.68	[42]
Meglumine antimoniate	97.00	4.10	[42]
Pentamidine	23.22	2.63	[43]

Given the significant limitations of current leishmaniasis treatments, including toxicity, high cost, and complex administration, there is a pressing need for alternative therapies that are safer, more affordable, and culturally accepted in endemic regions [40]. The inefficacy of conventional drugs, such as meglumine antimoniate, which demonstrated no meaningful antileishmanial activity against certain *Leishmania* species (IC_50_ > 100 µg/mL) and even allowed parasite proliferation during in vitro assays, further highlights this therapeutic gap [41]. In this scenario, natural products emerge as a promising alternative, offering bioactive compounds with potential antileishmanial properties, lower toxicity, and broader acceptance within traditional communities, thus supporting the development of innovative and sustainable treatment strategies.

Diverse chemical compounds were identified in Brazilian green propolis and *Baccharis dracunculifolia*, including diterpenes, flavonoids, and prenylated *p*‐coumaric acid derivatives. Both Brazilian green propolis and *B. dracunculifolia* exhibit comparable biological activities. In vitro leishmanicidal assays showed IC_50_ values of 45 and 49 µg/mL for *B. dracunculifolia* extract and GPE, respectively [29].

The results obtained in our study suggest that GPE may be more potent and effective (11.87 µg/mL), requiring lower quantities to achieve the same reduction in parasite viability, potentially offering significant advantages in terms of toxicity and treatment costs. UAE allowed for the retention of bioactive compounds, including phenolic compounds, phenolic acids, and flavonoids. Future studies plan to utilize biosynthesized AgNPs with GPE, representing an innovative approach to combat cutaneous leishmaniasis lesions.

### 3.9 Disk Diffusion Analysis

Antibacterial activity was confirmed by the disk diffusion method, where the zone of inhibition was measured using a digital caliper. AgNPs synthesized with NaBH_4_ (sodium borohydride, BH) and GPE exhibited no antimicrobial activity, as no zones of inhibition were observed when tested against *E. coli*, *S. aureus*, and *C. albicans*. In contrast, AgNP‐PRO showed antimicrobial activity at all concentrations and against all tested microorganisms (Table 4).

**TABLE 4:** Size of inhibition zones recorded from the disk diffusion method for *Escherichia coli*, *Staphylococcus aureus*, *Candida albicans* show in Figure 5, using GPE, AgNPs synthesized with NaBH_4_, and AgNPs synthesized with GPE using three different concentrations (1, 3, and 5 mM) of AgNO_3_ (AgNP1‐PRO, AgNP3‐PRO, and AgNP5‐PRO, respectively) in comparison to GPE alone.

	Inhibition zone (mm)		
Sample	*Escherichia coli*	*Staphylococcus aureus*	*Candida albicans*
AgNP1‐PRO	10	7	11
AgNP3‐PRO	9	7.5	8.5
AgNP5‐PRO	11	8	11
AgNPs‐NaBH_4_	0	0	0
GPE	0	0	0

As shown in Table 4, the presence of biological material is essential for the antimicrobial and antifungal activity of the nanoparticles, as AgNPs synthesized with NaBH₄ showed no inhibitory effect. The enhanced efficacy of AgNP‐PRO likely results from synergistic interactions between the metallic nanoparticles and secondary metabolites in GPE, contributing to microbial elimination. The mechanism underlying the antimicrobial activity of AgNPs remains, however, to be fully disclosed. Nevertheless, it is suggested that both silver ions and nanoparticles may interact with essential molecules for pathogen survival, such as proteins and DNA. Besides, AgNPs are known to induce the generation of reactive oxygen species (ROS), leading to apoptosis in microorganisms [26]. Spherical nanoparticles are often characterized by symmetrical SPR peaks, which suggest a uniform morphology. Besides, the lower the particle size, the higher the surface‐to‐ratio volume, which also promotes enhanced contact with the pathogens, increasing the antimicrobial effects [44, 45].

A comparative analysis between conventional AgNPs and AgNPs synthesized with propolis could provide valuable insights into the potential advantages of using green chemistry in nanoparticle production. Traditional chemical methods for synthesizing AgNPs often involve toxic reducing agents and stabilizers, which can pose environmental and health risks. In contrast, propolis, a natural substance with well‐known antimicrobial and antioxidant properties, offers a more eco‐friendly and sustainable approach to nanoparticle synthesis [46].

Santos et al. [47] evaluated the antimicrobial effects of pollen‐derived AgNPs against multidrug‐resistant bacteria. In the disk diffusion assay, AgNPs synthesized with pollen extract inhibited the growth of *E. coli* and *S. aureus*, whereas no antimicrobial activity was observed with the pollen extract alone. The inhibition zones (mean ± SD) were 11.59 ± 0.45 mm for *E. coli* and 8.33 ± 0.98 mm for *S. aureus*, highlighting the potential of pollen‐based AgNPs against resistant bacteria.

Daniel et al. [48] investigated the antimicrobial activity of TiO_2_ and AgNP films under visible light. Pure TiO_2_ films showed no inhibition, while composite films with AgNPs demonstrated increasing inhibition zones, that were proportional to the AgNP content. The largest inhibition zone (18.8 mm) was observed for the film with the highest silver concentration, with similar results (15 mm) comparable to gentamicin. The inhibition zone increased from 11.2 to 18.8 mm as the Ag content in the films increased, while pure AgNP films showed no defined inhibition.

### 3.10 HET‐CAM

The HET‐CAM provides crucial insights into the irritancy and safety profiles of AgNPs synthesized from GPE. This method is well‐established for assessing the initial toxicity and activity of drug delivery systems, bridging the gap between in vitro studies and in vivo [49, 50]. In our work, the HET‐CAM assay was employed to evaluate the risk of irritation of AgNP‐PRO at the vascular level of the chorioallantoic membrane.

**FIGURE 7:**
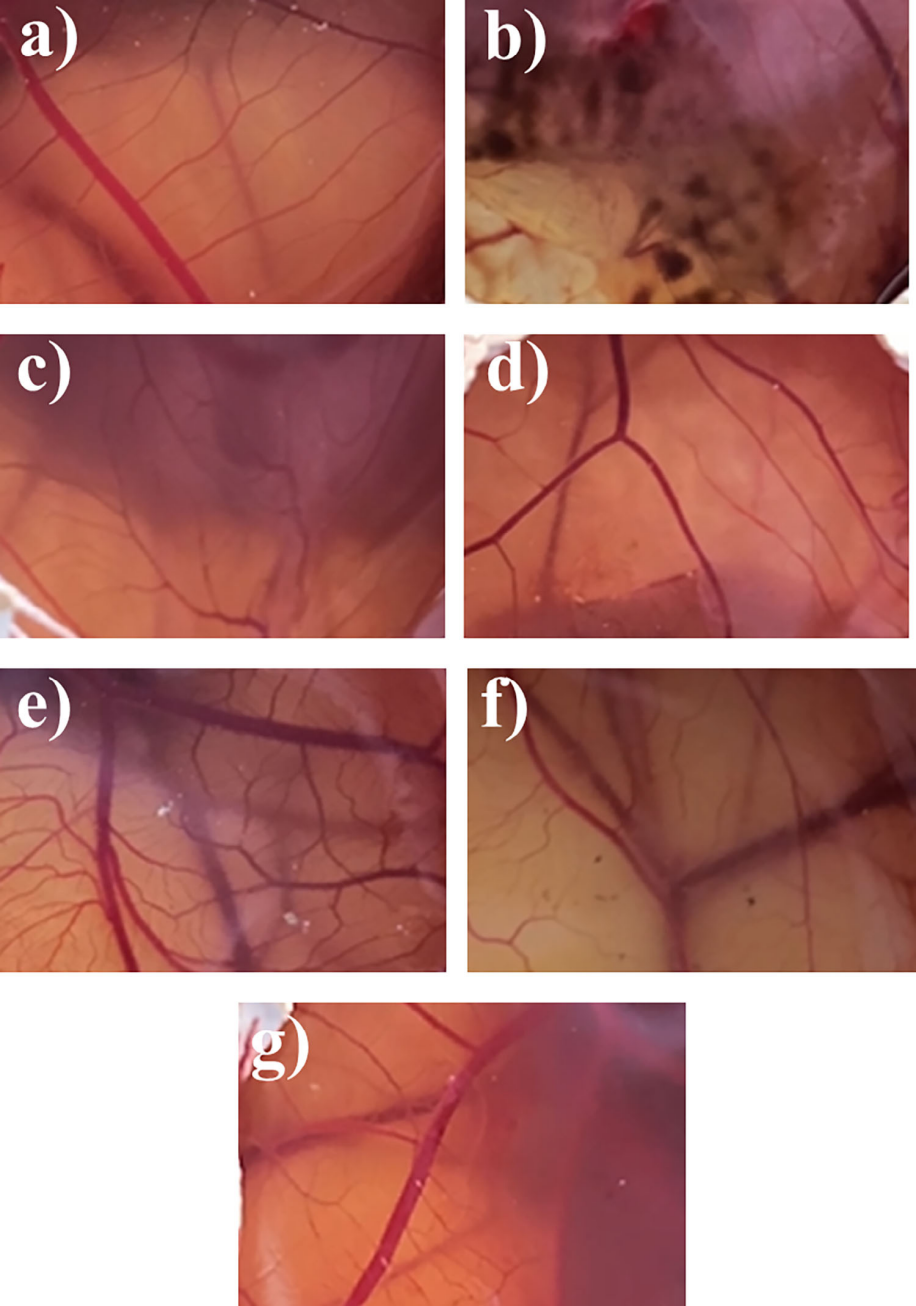
HET‐CAM assay. Endpoints for irritation were hemorrhage, coagulation, and vascular vasoconstriction. (a) 0.9% NaCl solution (negative control); (b) NaOH (positive control), (c) silver nanoparticles synthesized with sodium borohydride (BH); (d) green propolis extract (GPE); (e) silver nanoparticles synthesized with green propolis extract with 1 mM of AgNO_3_ (AgNP1‐PRO); (f) silver nanoparticles synthesized with green propolis extract with 3 mM of AgNO_3_ (AgNP3‐PRO); (g) silver nanoparticles synthesized with green propolis extract with 5 mM of AgNO_3_ (AgNP5‐PRO).

The HET‐CAM assay results confirmed that AgNP‐PRO does not induce any signs of irritation (Figure 7), supporting their safety profile and reinforcing their potential as effective leishmanicidal and antimicrobial agents for topical use. The HET‐CAM treated with particles bearing green propolis (Figure 7e–g) were comparable to that of negative control (a) and did not show the hemorrhage seen when HET‐CAM was treated with the particles synthesized with sodium borohydride (c). These findings align with previous studies on AgNPs synthesized via biological methods, such as those derived from *Clitoria ternatea*, which exhibited a particle size of 114 nm and a PDI of 0.45, indicating enough stability for topical administration [51]. Due to their potent antimicrobial properties and capacity to enhance wound healing, these nanoparticles are a promising candidate for the treatment of both acute and chronic wounds. In the study by Xie et al. [49], AgNPs were evaluated for their anti‐angiogenic efficacy using the HET‐CAM assay. No irritating effects were seen, supporting the potential of AgNPs for biomedical applications. In a study by Kosksi et al. [52], the authors reported that *Phlomis crinita* extract‐loaded polymeric nanoparticles exhibited a similar biocompatibility profile, further highlighting the suitability of the nanoparticles for dermatological and periocular applications.

## 4 Conclusions

The synthesis of AgNPs using GPE is a significant hallmark in the advancement of antimicrobial and antifungal research. The obtained nanoparticles (AgNPs‐PRO) were able to counteract the growth of *E. coli*, *S. aureus*, and *C. albicans* attributed to the synergistic interaction between metallic silver and the bioactive compounds in the natural extract (high content in flavonoids, phenolics, and caffeic acid derivatives), which were not recorded for AgNPs synthesized with NaBH_4_, nor with GPE alone. The results obtained with the HET‐CAM assay underscore the potential of AgNPs for a safe topical administration. This study highlights the potential for developing novel and sustainable antimicrobial and leishmanicidal strategies using a green chemistry approach. The different concentrations of AgNO_3_ showed a differential capacity to produce AgNPs. Our study suggests that chemical constituents present in GPE, such as artepillin C, may contribute to the reduction process of silver. These AgNPs‐PRO exhibit desirable characteristics, including a size around 100 nm, high stability, leishmanicidal and antimicrobial activities, and a nonirritation profile.

## Ethics Statement

The authors have nothing to report.

## Conflicts of Interest

The authors declare no conflicts of interest.

## Data Availability

Data will be made available from corresponding authors upon reasonable request.
